# Early seed priming with closely related *Bacillus* strains induces divergent physiological and defense responses in melon

**DOI:** 10.1093/hr/uhag053

**Published:** 2026-02-23

**Authors:** Luisa Carrégalo-Ríos, Carlos Molina-Santiago, María V Berlanga-Clavero, Daniel Petras, Jesús Hierrezuelo, Mónica Pineda, Juan M Alba, Antonio de Vicente, Matilde Barón-Ayala, Pieter C Dorrestein, Diego Romero

**Affiliations:** Instituto de Hortofruticultura Subtropical y Mediterránea “La Mayora,” Universidad de Málaga—Consejo Superior de Investigaciones Científicas (IHSM-UMA-CSIC), Málaga, Spain; Departamento de Microbiología, Universidad de Málaga, Bulevar Louis Pasteur 31 (Campus Universitario de Teatinos), Málaga 29071, Spain; Instituto de Hortofruticultura Subtropical y Mediterránea “La Mayora,” Universidad de Málaga—Consejo Superior de Investigaciones Científicas (IHSM-UMA-CSIC), Málaga, Spain; Departamento de Microbiología, Universidad de Málaga, Bulevar Louis Pasteur 31 (Campus Universitario de Teatinos), Málaga 29071, Spain; Instituto de Hortofruticultura Subtropical y Mediterránea “La Mayora,” Universidad de Málaga—Consejo Superior de Investigaciones Científicas (IHSM-UMA-CSIC), Málaga, Spain; Departamento de Microbiología, Universidad de Málaga, Bulevar Louis Pasteur 31 (Campus Universitario de Teatinos), Málaga 29071, Spain; Department of Biochemistry, University of California Riverside, Riverside, CA, USA; Instituto de Hortofruticultura Subtropical y Mediterránea “La Mayora,” Universidad de Málaga—Consejo Superior de Investigaciones Científicas (IHSM-UMA-CSIC), Málaga, Spain; Departamento de Microbiología, Universidad de Málaga, Bulevar Louis Pasteur 31 (Campus Universitario de Teatinos), Málaga 29071, Spain; Departamento de Estrés, Desarrollo y Señalización en Plantas, Estación Experimental del Zaidín, EEZ-CSIC, Profesor Albareda 1, 18008 Granada, Spain; Instituto de Hortofruticultura Subtropical y Mediterránea “La Mayora,” Universidad de Málaga—Consejo Superior de Investigaciones Científicas (IHSM-UMA-CSIC), Málaga, Spain; Departamento de Microbiología, Universidad de Málaga, Bulevar Louis Pasteur 31 (Campus Universitario de Teatinos), Málaga 29071, Spain; Departamento de Estrés, Desarrollo y Señalización en Plantas, Estación Experimental del Zaidín, EEZ-CSIC, Profesor Albareda 1, 18008 Granada, Spain; Collaborative Mass Spectrometry Innovation Center, University of California San Diego, La Jolla, CA, USA; Department of Biochemistry, University of California Riverside, Riverside, CA, USA; Instituto de Hortofruticultura Subtropical y Mediterránea “La Mayora,” Universidad de Málaga—Consejo Superior de Investigaciones Científicas (IHSM-UMA-CSIC), Málaga, Spain; Departamento de Microbiología, Universidad de Málaga, Bulevar Louis Pasteur 31 (Campus Universitario de Teatinos), Málaga 29071, Spain

## Abstract

Early microbial seed priming is conceived to improve crop resilience, yet it remains unclear whether plants can discriminate among closely related beneficial strains and integrate dose-dependent microbial cues. We primed melon (*Cucumis melo*) seeds with two phylogenetically similar *Bacillus* strains (*Bacillus subtilis* NCIB3610 and *B. velezensis* FZB42) and combined transcriptomic, metabolomic, and physiological analyses across development. Despite comparable colonization, the strains provoked contrasting host programs and distinct dose responses. *B. subtilis* promoted radicle elongation, chloroplastic starch storage, and drought tolerance regardless of inoculum level, together with l-tryptophan and palatinose accumulation. By contrast, *B. velezensis* displayed a clear dose effect: low inoculum sustained normal radicle growth, whereas high inoculum transiently repressed it, coinciding with suppression of allene oxide synthase, genes related to proteasome complex, and enrichment of flavonoids and glutathione in leaves. Chemical assays showed that radicle inhibition depends on the synergistic action of surfactin, produced by both strains, and bacillomycin D, an iturin-type lipopeptide specific to FZB42. This synergy explains the strain-specific lipopeptide repertoire to the dose-dependent growth response. Although their early trajectories diverged, both primings converged on enhanced aboveground stress resilience. 3610-primed plants restricted *Botrytis cinerea* via caffeic and rosmarinic acid accumulation, whereas FZB42-primed plants curtailed jasmonate-sensitive *Tetranychus urticae* mites through jasmonic acid pathway modulation. Our results demonstrate that melon perceives inoculum dose and microbial identity, translating them into distinct metabolic and defense programs that converge on stress resilience. These mechanistic insights (linking lipopeptide fingerprints, sentinel metabolites, and defense transcripts) provide a framework for precision seed treatments in horticultural crops.

## Introduction

Plants have coevolved with diverse microbial partners, developing interactions that range from mutualistic to pathogenic [1, 2]. The ability of plants to perceive and respond appropriately to microbial cues is central to maintaining this ecological balance, shaped by evolutionary selection of those interactions that promote survival and fitness of the plant holobiont [3]. While much attention has focused on defense responses to pathogens, less is known about how plants differentially interpret beneficial microbes, particularly those that are closely related yet exert a distinct function. Understanding how plants discriminate among beneficial microorganisms is key to unraveling the ecological and evolutionary dynamics of plant–microbiome interactions. Microbial associations initiated at the seed stage are among the earliest interactions in the plant life cycle [4, 5] and can influence development and stress responses across tissues and life stages [6]. These early interactions offer a unique opportunity to explore how plants integrate microbial information to shape their adaptive responses throughout their development.

*Bacillus* species are widely used as plant growth-promoting rhizobacteria [7, 8]; however, most studies treat these microbes as functionally equivalent, focusing on their general effects on yield or pathogen suppression [9]. Much of the literature focuses on a single strain and its ability to promote growth or enhance stress tolerance [10], overlooking the underlying recognition mechanisms that might lead to distinct yet beneficial responses that could converge on a similar phenotype. From the plant’s perspective, mutualism is not a binary state but a flexible interaction shaped by functional compatibility. Different beneficial microbes, even those that are phylogenetically close, may engage the host in distinct ways [11, 12]. The extent to which a plant can modulate its metabolic and defense programs in response to strain-specific interactions remains largely unexplored.

In this study, we use early seed priming with two closely related *Bacillus* strains, *B. subtilis* NCIB3610 and *B. velezensis* FZB42, as a tool to probe how the plant host modulates its adaptive programming in a mutualistic context. Despite similar colonization efficiency, the two strains trigger distinct developmental, metabolic, and defensive trajectories in *Cucumis melo*. By integrating transcriptomic, metabolomic, anatomical and physiological data, we show that the plant does not merely respond to microbial presence, but actively differentiates between beneficial partners, demonstrating that mutualism itself can take multiple functional forms, shaped by microbial identity.

## Results

### Dose-dependent root developmental responses to *Bacillus* seed treatments

**Figure 1:**
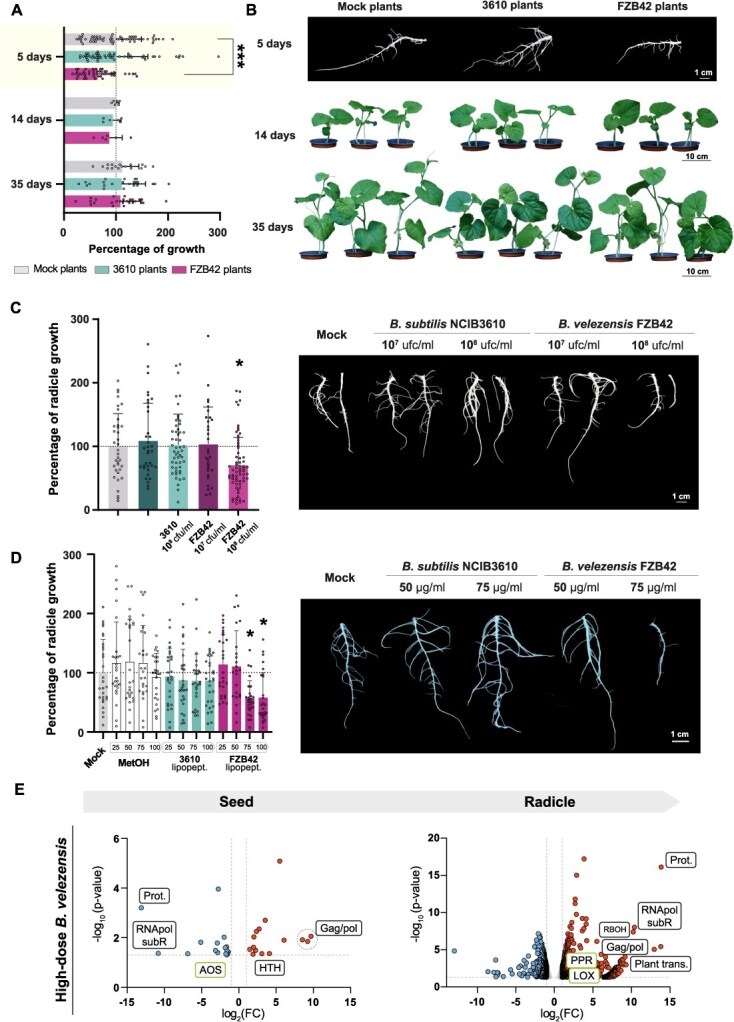
Early seed priming with *B. velezensis* delays development despite exhibiting similar ecological traits to *B. subtilis* on melon seeds. **A** Growth progression (%) of plants grown from seeds treated with *B. subtilis* NCIB3610 (blue) or *B. velezensis* FZB42 (purple), relative to mock-treated controls (gray, distilled water). Growth was normalized to the average radicle area at 5 days posttreatment (dpt) (highlighted, yellow background) or average fresh weight at 14 and 35 dpt (white background) of mock plants (dashed line = 100%). Mean ± SD. Statistical significance was assessed using one-way ANOVA with Dunnett’s *post hoc* test (^***^P < 0.001). **B** Representative images of radicles (5 dpt) and whole plants (14 and 35 dpt) derived from mock- and *Bacillus*-treated seeds. **C** Left: Radicle development (%) 5 days after seed inoculation with *B. subtilis* or *B. velezensis* at low (10^7^ CFU ml^−1^) or high (10^8^ CFU ml^−1^) bacterial densities. Data are normalized to mock controls (dashed line = 100%. Mean ± SD. Statistical analysis as above (^*^P < 0.05). Right: Representative radicles from each treatment. Scale bar = 1 cm. **D** Left: Radicle development (%) 5 dpt with crude lipopeptide extracts from *B. subtilis* or *B. velezensis* (25–100 μg/ml). Methanol control included. Data normalized to mock controls (dashed line = 100%. Mean ± SD. One-way ANOVA with Dunnett’s *post hoc* test (^*^P < 0.05). Right: Representative radicles per condition. Scale bar = 1 cm. **E** Volcano plot showing differentially expressed genes (DEGs) in seeds treated with high-dose *B. velezensis* and their corresponding radicles. *P* values calculated using Fisher’s method across edgeR and DESeq2 values. Thresholds for significance (horizontal = *P*, vertical = fold change) are shown as dashed lines. Green labels indicate DEGs shared with low inoculum dataset (AOS, PPR and LOX). Selected genes are annotated for retrotransposon activity, oxidative stress, and plant defense (full data in Table S1).

Melon seeds treated with *B. velezensis* FZB42 exhibited smaller radicles compared to those treated with either *B. subtilis* NCIB3610 or distilled water (referred to as ‘mock plants’ hereafter) after 5 days of growth (Fig. 1A and B).

Both bacterial strains were able to colonize the seed and persisted during radicle development, reaching 100% sporulation within 48 hours (Fig. S1A). *Bacillus subtilis and B. velezensis* were also recovered from adult plant roots, with sporulation rates of 31.2% and 57.3%, respectively (Fig. S1B), highlighting their successful establishment as root colonizers. To understand the observed differences in radicle growth, various bacterial inoculum concentrations were tested. A lower inoculum concentration of *B. velezensis* [10^7^ colony-forming unit (CFU) per milliliter instead of the initial 10^8^ CFU ml^−1^] restored radicle growth to mock plant phenotype, while *B. subtilis* consistently showed a trend toward growth promotion at both doses tested (Fig. 1C). The radicle growth repression induced by *B. velezensis* at higher inoculum concentration was also observed in *Arabidopsis thaliana*, a species phylogenetically distant from melon (Fig. S1C). Importantly, the early repression of radicle growth did not translate into detrimental consequences during later developmental stages, as all plants reached the same developmental stage by 35 days (Fig. 1A and B).

Previous report indicated that fengycin contributes to oxidative stress and tissue damage when applied to melon seeds, promoting overgrowth of adult plants [13]. In addition to fengycin, *B. velezensis* FZB42, but not *B. subtilis* NCIB3610, produces bacillomycin D, an iturin-like lipopeptide (Fig. S1D) [14], which led us to hypothesize a potential negative effect of this compound on root growth. Supporting this idea, crude lipopeptide extracts from *B. velezensis* cultures induced significant radicle growth inhibition at 75 and 100 μg/ml (Fig. 1D), whereas lower concentrations (25 and 50 μg/ml) did not reproduce the effect. In contrast, treatments with purified lipopeptides did not inhibit radicle growth. Bacillomycin D (20 μM) even promoted growth, both alone and in combination with either fengycin (20 μM) and surfactin (20 μM). Interestingly, surfactin alone showed a tendency toward repression. Notably, the growth-promoting effects of bacillomycin D and fengycin were lost when surfactin was included in combination with both lipopeptides at the same time (Fig. S1E). These results indicate that radicle growth repression emerges from the combined activity of the full lipopeptide mixture rather than from direct synergy between purified individual molecules.

We previously reported that initial radicle growth does not necessarily predict adult plant development or resistance to biotic stress. Nevertheless, *B. subtilis* triggered changes in the dynamics of triacylglycerides after differential targeting of seed oil bodies and lipid catabolism optimization, as shown by transcriptomic analysis [13]. Since all groups reached similar developmental stages over time, we hypothesized that seeds treated with a higher inoculum of *B. velezensis* (‘FZB42 plants’) undergo adaptive developmental reprogramming, favoring long-term benefits over early growth. Consistent with this hypothesis, transcriptomic analysis performed immediately after high-dose *B. velezensis* seed treatment revealed strong upregulation of endogenous retrovirus and retrotransposon-related genes, particularly those encoding Gag/Pol proteins (Fig. 1E), together with repression of proteasome components (Table S1). Radicle transcriptomes from high-inoculum treatments exhibited a global increase in transcriptional activity, as reflected by the broader distribution and higher number of differentially expressed genes in the volcano plots compared with the low-inoculum treatment (Fig. 1E; Fig. S2A). Transposable elements are known to influence genome architecture and gene regulation in response to environmental stimuli [15], providing a potential regulatory context for this widespread transcriptional activation. In parallel, allene oxide synthase (*AOS*), a key enzyme in jasmonic acid (JA) biosynthesis [16], was downregulated in a dose-dependent manner (log_2_FC = −1.7 at low dose and − 2.9 at high dose; Table S1). In the context of the JAZ repressor model, where low JA-Ile levels maintain a primed defense state [17], this transcriptional profile is consistent with a lowered threshold for subsequent defense activation. Gene ontology enrichment analysis further revealed overrepresentation of terms associated with oxidative stress and redox-related processes (Fig. S2B and C), indicating that early seed treatment induces a transient stress-associated transcriptional state.

### Aboveground physiological and anatomical adaptations following *Bacillus* priming

**Figure 2:**
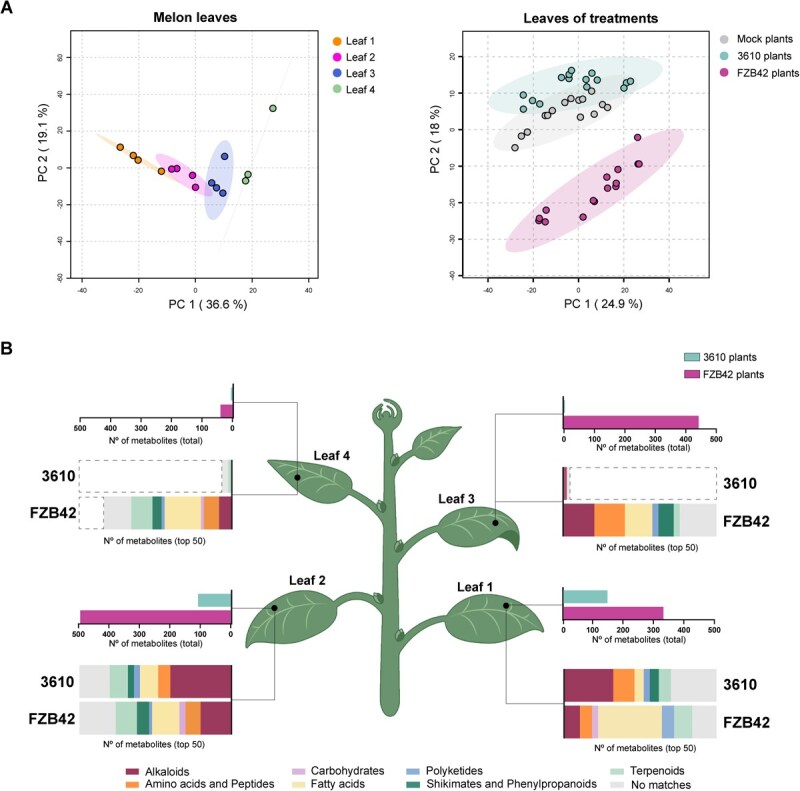
*Bacillus* strains trigger distinct patterns of metabolite accumulation in melon leaves. **A** Principal component analysis (PCA) of metabolomic profiles from melon leaves. Left: Clustering based on leaf developmental stage in mock-treated plants. Right: Clustering based on seed treatment (*B. subtilis* NCIB3610, *B. velezensis* FZB42, and mock). Percentage of variance explained by each principal component is indicated on the axes. **B** Metabolite accumulation summary based on volcano plot analysis comparing each *Bacillus* treatment to mock. Upper graphs within one leaf: number of significantly accumulated metabolites in 3610-treated (blue, upper bar within graph) and FZB42-treated (pink, lower bar within graph) plants. Lower panels within one leaf: chemical classification (NPC#pathway) of the top 50 discriminant metabolites with significant variation (log₂FC > 2 and FDR-adjusted *P* < 0.05).

Given the transcriptomic and phenotypic differences observed during seed and radicle stages, we analyzed metabolic profiles in adult leaves. Metabolomic analysis across leaf sections from mock plants revealed age-dependent variations, as well as metabolite accumulation influenced by seed treatment (Fig. 2A). The most pronounced changes were observed in older leaves of FZB42 plants (Fig. S3) with between 300 and 500 differentially accumulated metabolites in the second and third leaves. Younger leaves showed fewer than 100 accumulated metabolites. *B. subtilis*-treated plants (‘3610 plants’) showed a similar gradient, with older leaves accumulating more metabolites than younger ones. Chemical classification of the top 50 metabolites differentially accumulated by treatment and leaf age revealed distinct signatures: FZB42 plants accumulated fatty acids, particularly in the oldest leaf, while 3610 plants exhibited increased levels in alkaloids, including tryptophan derivatives (Fig. 2B). Glycerolipids and glycerophospholipids were enriched in FZB42 plants, suggesting a shift in lipid metabolism (Fig. S4A).

**Figure 3:**
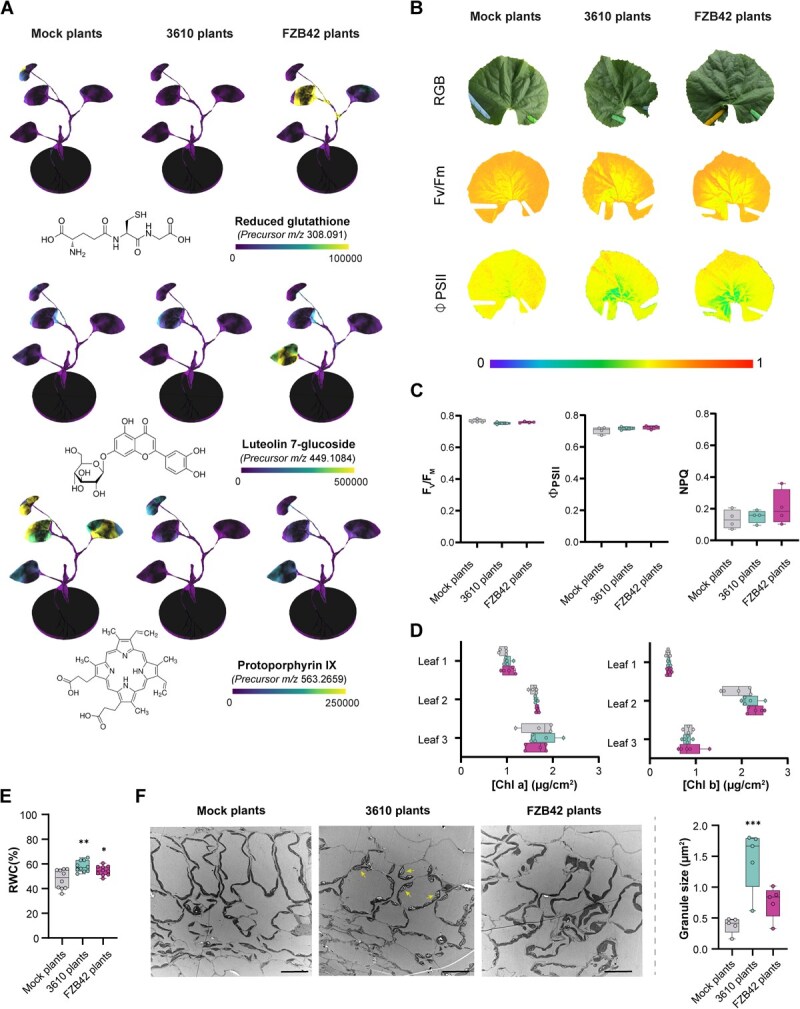
*Bacillus*-primed plants maintain photosynthetic capacity while showing adaptive traits, including oxidative stress modulation and increased water retention. **A** 3D spatial distribution of selected metabolites in leaves of mock- and *Bacillus*-treated plants, identified by spectral match: reduced glutathione (top), luteolin 7-glucoside (middle), and protoporphyrin IX (bottom). **B** Representative images showing RGB, maximum quantum efficiency of PSII (F_V_/F_M_), and effective quantum yield of PSII (Φ_PSII_) in steady state in leaves from each treatment group. **C** Quantitative values of F_V_/F_M_ (left), Φ_PSII_ (middle), and nonphotochemical quenching (NPQ; right) from leaves of mock- and *Bacillus*-treated plants. Box plots show median (horizontal line) and range (whiskers). **D** Chlorophyll *a* content (μg cm^−2^) (left) and chlorophyll *b* content (μg cm^−2^) (right) by leaf developmental stage per plant group. **E** Relative water content (RWC) of leaves from mock- and *Bacillus*-treated plants. Box and whisker plot. One-way ANOVA with Dunnett’s *post hoc* test (^*^P < 0.5; ^**^P < 0.01). **F** Left: TEM images of thin leaf sections showing increased starch accumulation in chloroplasts of *B. subtilis*-primed plants. Scale bar = 20 μm. Right: granule size (μm^2^) per plant group. Box and whisker plots based on five slices from three biological replicates. One-way ANOVA with Dunnett’s *post hoc* test (^***^P < 0.001).

All plant groups exhibited a shared healthy phenotype at the adult plant stage; however, the transcriptomic (at seed and radicle level) and metabolic differences (leaves of adult plants) between treatments prompted us to investigate potential divergences in physiological and anatomical outcomes. Further refinement of feature-based molecular networking of the collected metabolome data identified three metabolites of interest: reduced glutathione (GSH), significantly accumulated in leaves of FZB42 plants; luteolin 7-glucoside, a flavonoid with potent antioxidant activity [18], mainly accumulated in leaves of FZB42 plants but also present in 3610 plants; and protoporphyrin IX, accumulated in mock plants and not associated with either of the bacterial treatments (Fig. 3A). To investigate whether the reduced accumulation of a chlorophyll precursor in treated plants might compromise photosynthesis, a fundamental process for plant growth [19], physiological assays were conducted. Photosynthetic performance of the second leaf was evaluated at 35 days after sowing (adult plants) by measuring the maximum quantum yield of photosystem II (PSII) (F_V_/F_M_) and effective quantum yield of PSII at steady state (Φ_PSII_). In plants grown from *Bacillus*-treated seeds, imaging analysis revealed a slight reduction in photosynthetic efficiency at the leaf base near the petiole, particularly in terms of Φ_PSII_ compared to mock plants. However, this decrease was offset by increased photosynthetic activity in other leaf regions (Fig. 3B). Indeed, when averaged across the entire leaf surface, 3610 and FZB42 plants exhibited no significant differences in Φ_PSII_ or F_V_/F_M_ compared to mock plants, demonstrating that the overall photosynthetic yield of melon plants was not affected by seed treatment (Fig. 3C). Similarly, nonphotochemical quenching at the steady-state NPQ, representing energy not used in photosynthesis and dissipated as heat [20], remained invariable between treatments (Fig. 3C). Photosynthesis is tightly regulated to prevent the accumulation of reactive intermediates that could induce oxidative stress [21]. Given the evidence of an elevated oxidative state in plants grown from *Bacillus*-treated seeds, marked by latent metabolic imprints in GSH and luteolin 7-glucoside accumulation, our results suggest an optimization of this fundamental metabolic process. Moreover, despite exhibiting less accumulation of protoporphyrin IX, no differences in chlorophyll *a* and *b* content were observed in 3610 and FZB42 plants (Fig. 3D). Finally, the greater water content within the leaves of 3610 and FZB42 plants compared to mock plants (Fig. 3E) further supports this hypothesis, in line with the well-established dependence of photosynthetic efficiency on leaf water status [22]. In light of these results, we hypothesize that 3610 and FZB42 plants might count with an ameliorated photosynthetic process where the same needs are met with fewer content of photosynthesis intermediates.

To visualize anatomical changes derived from seed treatment, second leaf sections of adult plants were analyzed by transmission electron microscopy (TEM) and scanning electron microscopy (SEM). Plants grown from seeds treated with *B. subtilis* exhibit increased starch accumulation inside chloroplasts, as shown by TEM analysis of thin leaf sections (Fig. 3F). Starch, a complex carbohydrate polymer commonly found in plant cells, accumulates during the day under light and breaks down at night to provide sugars for metabolism and growth [23]. Since all samples were collected at the same time of day, any differences observed between plant groups likely reflect variations in either the accumulation or degradation patterns of reserve carbohydrates. Based on nontargeted metabolomics data, palatinose (also known as isomaltulose; α-d-glucopyranosyl-1,2-d-fructofuranose) [24] accumulates in the oldest leaf of 3610 plants (Fig. S4B). This naturally occurring analog of sucrose has been reported to stimulate sucrose degradation and starch synthesis [25], suggesting that carbon flux in 3610 plants may be promoted toward starch accumulation. In accordance with these findings, *Bacillus*-treated plants showed an induction of the expression of Sucrose synthase 2 (SucS2), a key enzyme catalyzing the reversible conversion of sucrose and UDP into UDP-glucose and fructose (ref), thus providing substrates for starch biosynthesis (Fig. S4C). This mechanism likely facilitates the establishment of a carbohydrate reserve, ensuring both a readily available energy source for future metabolic demands and a source of soluble sugars that may function as osmoprotectants during environmental challenges [26]. Compared to mock and 3610 plants, FZB42 plants exhibited a higher stomatal density, but only on the adaxial surface of the second leaf at the adult plant stage (Fig. S5A and B). Additionally, FZB42 plants showed a tendency to maintain stomata open, suggesting an elevated leaf transpiration rate. Consistently, leaf surface temperature, inversely correlated with leaf transpiration and evaporation [27], was significantly lower in FZB42 plants compared to the other treatments (Fig. S5C and D).

### Improved abiotic stress resilience linked to *Bacillus*-mediated seed priming

**Figure 4:**
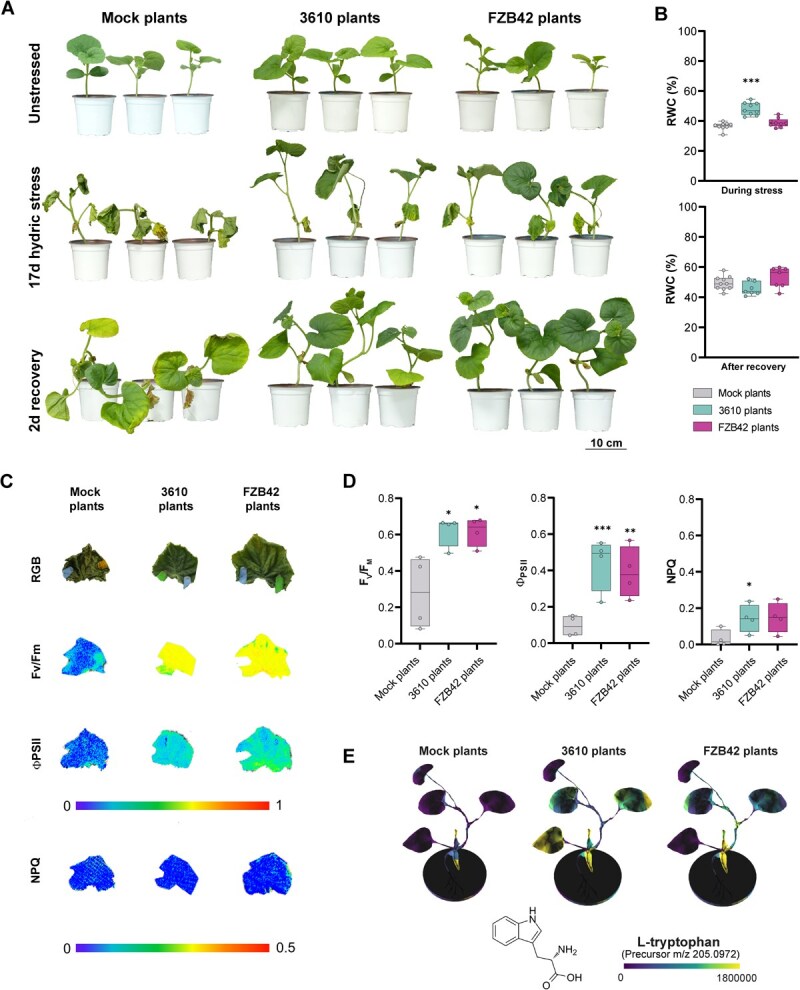
*Bacillus subtilis* seed priming enhances drought resilience in melon plants. **A** Representative image of mock-, *B. subtilis*-, and *B. velezensis*-primed plants under three conditions: well-watered (top), after 17 days of drought (middle), and 2 days post-rewatering (bottom). **B** Relative water content (RWC) of leaves from mock- and *Bacillus*-treated plants under drought and after rewatering. Box and whisker plot. One-way ANOVA with Tukey’s *post hoc* test (^***^P < 0.001). **C** RGB images, F_V_/F_M_, Φ_PSII_ (steady state), and NPQ (steady state) measured 2 days after rewatering in leaves from each treatment group. **D** Quantitative values of F_V_/F_M_ (left), Φ_PSII_ (middle), and NPQ (right). One-way ANOVA with Dunnett’s *post hoc* test (^*^P < 0.05; ^**^P < 0.01; ^***^P < 0.001). **E** Spatial distribution of l-tryptophan abundance (spectral match) in 3D models of mock- and *Bacillus*-treated plants.

To explore potential landscapes where the aforementioned physiological and metabolic differences may be significant, we assessed plant responses to various abiotic and biotic stressors. Given the increased water content in *Bacillus*-treated plants (Fig. 3D) and the potential role of starch in osmotic adjustment in 3610 plants (Fig. 3E), we investigated plant drought response. After 17 days without irrigation, 3610 and FZB42 plants appeared visibly healthier than mock plants, with 3610 plants exhibiting the highest relative leaf water content (RWC) of all plant groups during stress conditions. Upon rewatering, 3610 and FZB42 plants recovered faster than mock plants; however, no significant differences in RWC were recorded at this stage (Fig. 4B). To further investigate the physiological basis of this response, we assessed photosynthetic performance during recovery by measuring F_V_/F_M_ and steady-state Φ_PSII_ across the entire second leaf surface (Fig. 4C and D). Compared to well-watered plants (Fig. 3B and C), all groups exhibited reduced photosynthetic activity, confirming that water deprivation severely impacted primary metabolism. However, 3610 and FZB42 plants showed significantly higher F_V_/F_M_ and steady-state Φ_PSII_ values than mock plants (Fig. 4D), suggesting that only the latter suffered irreversible damage to the chloroplast electron transport chain. Consistently, steady-state NPQ, a key photoprotective mechanism against photooxidation in the thylakoid membranes [28], remained significantly elevated in *Bacillus*-treated plants in comparison to mock plants (Fig. 4D). Along with higher RWC and starch accumulation, l-tryptophan was significantly accumulated in both younger and older leaves of nonstressed 3610 plants, as well as in the third leaf of FZB42 plants (Fig. 4E). This amino acid may confer additional protection against desiccation, given that exogenous l-tryptophan treatment has been reported to alleviate hydric stress [29].

### Species-specific priming against biotic stressors in adult plants

**Figure 5:**
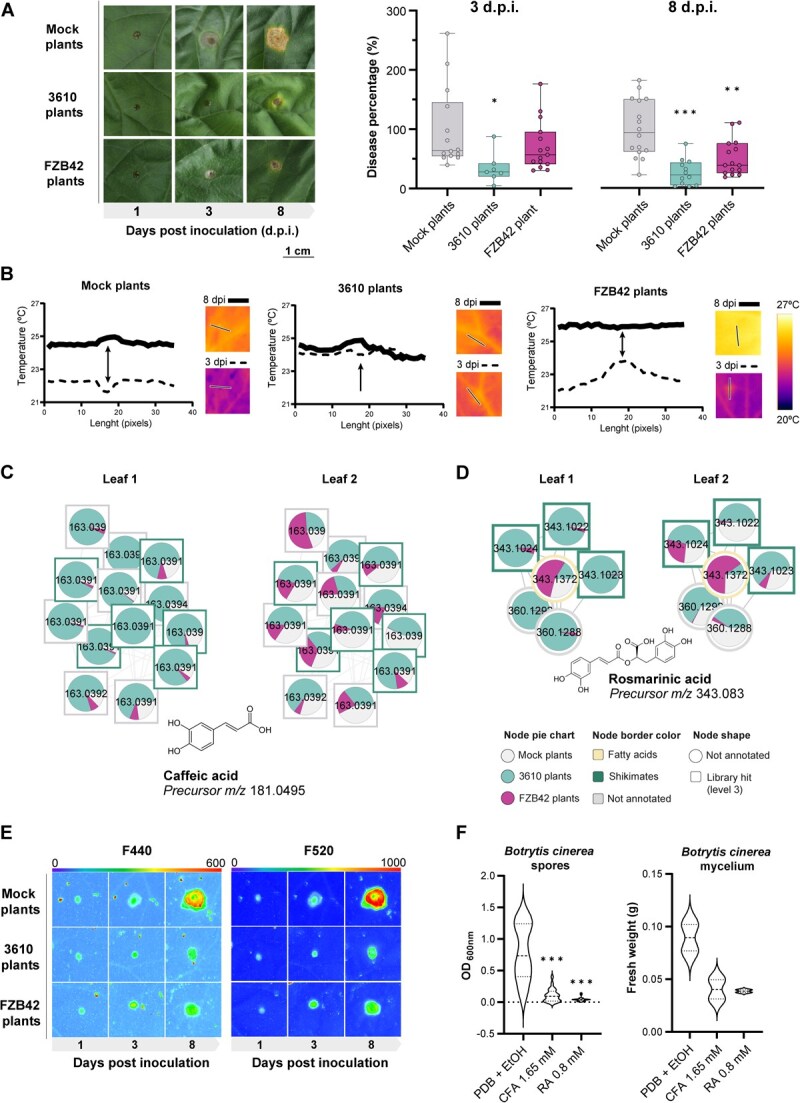
Seed priming with *Bacillus* strains enhances phenolic accumulation and resistance to *B. cinerea* in melon. **A** Left: Necrotic lesions in leaves from mock-, *B. subtilis*-, and *B. velezensis*-treated plants, 1–8 days after *B. cinerea* inoculation. Right: Lesion area quantification normalized to mock-treated controls at 3 and 8 dpi. Box-and-whisker plots. One-way ANOVA with Dunnett’s *post hoc* test (^*^P < 0.05; ^**^P < 0.01; ^***^P < 0.001). **B** Leaf surface temperature profiles (35-pixel transect) across lesions and surrounding tissue. Central inoculation point indicated with an arrow. **C, D** Molecular family networks of caffeic acid (CFA) (C) and rosmarinic acid (RA) (D) and related phenolics differentially accumulated in leaves from mock- and *Bacillus*-treated plants. Pie charts show mean relative abundance. Node shape: identification level [30]; border color: chemical class (NPC#pathway). **E** Fluorescence imaging (F440 and F520) of cell wall-bound phenolics at the inoculation site and adjacent areas. **F** Left: Growth inhibition of *B. cinerea* spores treated with CFA or RA *in vitro* (48-well assay, 3 days). Right: Mycelial growth inhibition with CFA and RA (24 h). Control = PDB + 0.8% ethanol. One-way ANOVA with Dunnett’s *post hoc* test (^***^P < 0.001).

We have previously noted that fengycin-treated seeds confer immunity to the adult plant against the aboveground pathogen *Botrytis cinerea* [13]. To determine whether this response is conserved among *Bacillus* species, we conducted infection assays by inoculating the second leaf of all plant groups with a spore suspension of this phytopathogen. The size of necrotic lesions induced by the fungus in 3610 plants was significantly smaller than that of mock or FZB42 plants 3 days postinoculation (d.p.i.). However, by the end of the experiment, at 8 dpi, the size of the necrotic lesions was significantly smaller in both 3610 and FZB42 plants (Fig. 5A). To investigate the physiological underpinnings of this response, we analyzed leaf transpiration patterns following fungal infection by thermography imaging, as they are shown to be reduced in *B. cinerea*-infected melon plants [31] (Fig. 5B). *Botrytis cinerea* altered whole-leaf transpiration patterns across all treatments compared to noninfected leaves by 3 dpi, based on the reduction in average leaf temperature (Fig. 5B; Fig. S5C and D). Temperature decreases were most pronounced in mock plants (−2.2°C), followed by FZB42 plants (−1.7°C) and 3610 plants (−1.4°C). By 8 dpi, leaf temperatures returned to preinfection levels in all groups except FZB42 plants, whose temperature exceeded those values. To assess localized effects of *B. cinerea* infection and its spatial spread, transects (~12 mm; 35 pixels) centered on the inoculation sites were analyzed from thermal images. Temperature values for each pixel have been plotted on the corresponding profiles (Fig. 5B).

At 3 dpi, mock plants displayed the lowest temperatures across transects, particularly at the inoculation point. By 8 dpi, overall transect temperatures increased, with the most pronounced rise occurring at the inoculation point. In contrast, 3610 plants exhibited higher, more uniform temperatures across transects at 3 dpi, with minimal fluctuations at 8 dpi. It is worth noting that 3610 plants at 3 dpi reached temperature profiles similar to those of mock plants at 8 dpi, suggesting an earlier detection of the pathogen. FZB42 plants, however, showed an early localized temperature increase at 3 dpi at the inoculation site, while surrounding areas remained comparable to mock plants. By 8 dpi, FZB42 plants exhibited the highest and most uniform temperatures across the analyzed transect, with no apparent temperature gradients.

To further characterize *B. cinerea* infection dynamics in 3610 and FZB42 plants, where disease percentage was significantly reduced, specific metabolites potentially involved in defense responses were analyzed. Nontargeted metabolomics identified significant accumulation of caffeic acid (CFA) and rosmarinic acid (RA; an ester of caffeic acid and 3,4-dihydroxyphenyllactic acid) in older leaves of 3610 plants (Fig. 5C and D; Table S2). These metabolites may localize within the apoplast or become esterified to the cell wall. To clarify their anatomical distribution, melon plants were illuminated with UV light, and the fluorescence emitted by phenolic compounds covalently bonded [32] to the cell wall was recorded in the blue (F440) and green (F520) regions of the spectrum. Blue-green fluorescence (BGF) emission was measured in the second leaf of adult-stage healthy melon plants (Fig. S6A and B). No significant differences were observed in F440 and F520 emission either in the spatial pattern or averaged over the whole leaf across the three treatments in the absence of pathogen inoculation. On the other hand, BGF emission of *B. cinerea*-inoculated leaves was also recorded to test whether the compounds remained soluble or esterified by enhancing the known increase in F440 and F520 emissions of the affected areas [31] (Fig. 5E). At 3 dpi, fungal lesions on mock and FZB42 plants could be visualized as a spot with higher F440 and F520 emission than the surrounding areas. Interestingly, in 3610 plants, the *B. cinerea*-affected area did not increase in size at 3 dpi. By the final measurement, the affected area expanded in all treatments, as indicated by higher F440 and F520 emissions. Mock plants showed the highest F440 and F520 emissions and the largest affected area, followed by FZB42 plants and finally 3610 plants, which showed the lowest BGF emission and the narrowest affected area. When *B. cinerea* spores were inoculated with commercial caffeic acid and rosmarinic acid, their growth was inhibited compared to nontreated spores. Similarly, treatment of already germinated spores with these compounds arrested further mycelial growth (Fig. 5F). Interestingly, these compounds did not impair growth or biofilm formation of either *B. subtilis* or *B. velezensis* when tested *in vitro* (Fig. S6C and D). These findings indicate that soluble phenolic compounds accumulated in leaves due to *Bacillus* treatment of the seeds are cytotoxic on *B. cinerea* likely preventing fungal spread to surrounding areas and, thus, reducing necrotic lesion size.

**Figure 6:**
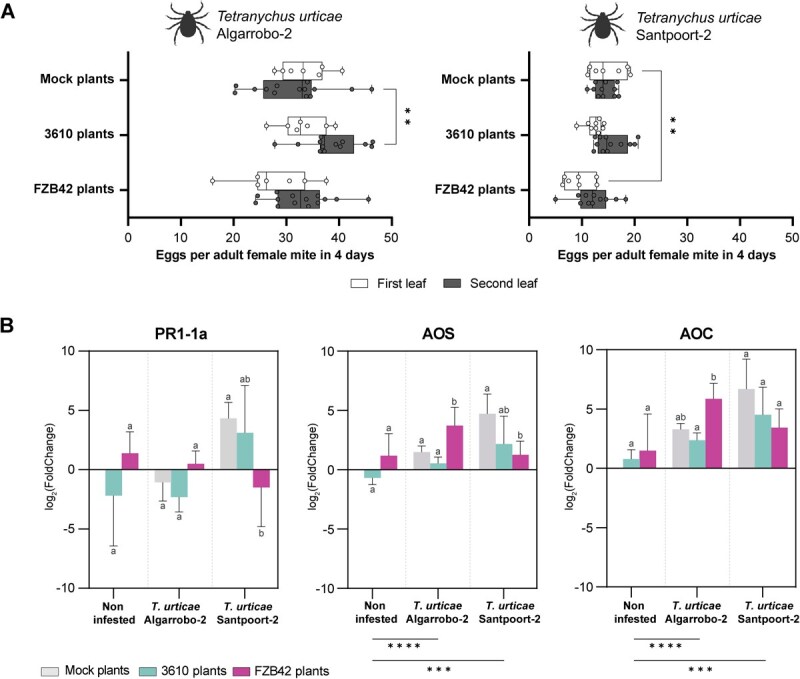
Seed priming with *Bacillus velezensis* alters plant–herbivore interactions, impairing performance of JA-sensitive spider mites. **A** Reproductive performance (number of eggs/female in 4 days) of *T. urticae* and *T. urticae* Santpoort-2 on first and second leaves. Box-and-whisker plots show distribution across replicates (dots = individual values). Two-way ANOVA followed by a *post hoc* Tukey’s test (^**^P < 0.001). **B** Relative transcript abundance (log₂FC) of defense-related genes in second leaves of mock- and *Bacillus*-treated plants under noninfested or mite-infested conditions. Genes include PR1-1a (SA pathway), *AOS* and *AOC* (JA pathway). Mean ± SD. Values were normalized to the housekeeping gene *actin*-7 and expressed relative to noninfested mock plants while all statistical analyses were performed on normalized ΔCt data. Two-way ANOVA followed by Tukey’s *post hoc* tests. Letters indicate significant differences among bacterial treatments within infestation scenarios, while brackets denote significant effects between infestation scenarios (^**^P < 0.001; ^****^P < 0.0001).

Previous studies have associated the accumulation of plant specialized metabolites, including terpenoids, green-leaf volatiles, and aromatic compounds, with defense against herbivores [33]. While terpenoids play a crucial role in attracting natural enemies both above and below ground [34], alkaloids are well known for their potent toxicity against various organisms, including arthropods, by interacting with DNA, membranes, and enzymes [35]. In addition, chlorogenic acid has been described as an important metabolite in defense against herbivores [36]. Given the accumulation of these metabolites in plants grown from *Bacillus*-treated seeds (Fig. 2C; Fig. S4A and B), we conducted experiments to assess whether this observation translates into enhanced herbivore resistance. We examined to what extent the metabolic status of leaves influences herbivore behavior using two *Tetranychus urticae* strains: Algarrobo-2, a broad host range strain [37], and Santpoort-2, previously described as susceptible to induced defenses [38]. Indeed, the reproductive performance of *T. urticae* Santpoort-2 was significantly reduced on plants grown from *B. velezensis*-treated seeds (Fig. 6A). To probe underlying signaling, we measured transcript abundance of salicylic acid (SA)- and JA-related markers by quantitative reverse-transcriptase PCR (qRT-PCR). Plants grown from seeds treated with *B. velezensis* tended to upregulate the SA marker *PR1-1a* [39] in the absence of herbivores, whereas JA biosynthetic genes (*AOS*, *AOC*) [40] were induced upon mite presence (Fig. 6B), thus downregulating *PR1-1a*, probably via the crosstalk between JA and SA pathways [41]. Notably, infestation with Algarrobo-2 produced the strongest induction of *AOS* and *AOC* in FZB42 plants, yet this pronounced transcriptional response did not reduce Algarrobo-2 oviposition within the 4-day assay. In contrast, Santpoort-2 performance was impaired on FZB42 plants despite a comparatively weaker induction of JA biosynthetic transcripts. Together, these data suggest that seed treatment with *B. subtilis* 3610 and *B. velezensis* FZB42 differentially modulates plant–mite interactions: *B. velezensis* elicits a strong JA transcriptional response to Algarrobo-2 that is insufficient to limit a broadly adapted strain, while conferring functionally effective resistance against a JA-sensitive strain.

## Discussion

By comparing two phylogenetically related beneficial bacteria applied at the seed stage, we demonstrate that early microbial priming leads to distinct physiological and metabolic programs in *C. melo*. Despite similar colonization efficiency and persistence, *B. subtilis* and *B. velezensis* induced divergent developmental dynamics, leaf metabolite profiles, and stress-related responses. These results underscore the plant’s capacity to functionally differentiate between beneficial microbial partners and tailor its physiological trajectory accordingly.

Notably, *B. velezensis* induced a transient delay in radicle emergence, particularly at higher doses, suggesting a threshold-dependent effect on early growth. Previous work has shown that structural variants of lipopeptides produced by *Bacillus* strains can modulate biological activity and host perception, potentially accounting for the delay observed [42]. Despite early modulation of growth, plants treated with *B. velezensis* reached developmental stages comparable to controls, indicating that early reprogramming does not compromise long-term performance, in line with the current paradigm highlighting the existence of a recovery phase in which plants either reset or consolidate stress memory [43]. Transcriptomic data revealed upregulation of retrotransposon-related genes and repression of proteasome components in seeds treated with *B. velezensis*, suggesting a regulatory state in which genomic flexibility is increased while proteolytic activity is restrained. Importantly, this transcriptional profile was accompanied by a marked increase in global transcriptional activity and enrichment of oxidative stress-related processes, indicating that high-dose seed treatment induces a transient stress-associated and highly transcriptionally active state at early developmental stages. Such a physiological context may facilitate large-scale regulatory reorganization, including transposon activation and hormonal pathway modulation, thereby contributing to early adjustments in developmental plasticity. While our data do not directly address epigenetic mechanisms, parallels with stress memory upon pathogenic infection [44, 45] and mutualistic fungal interactions [2, 46] raise compelling questions about the role of chromatin-level reprogramming in bacterial priming.

At the whole-plant level, *Bacillus*-treated plants displayed species-specific metabolite accumulation patterns in leaves. While both strains maintained photosynthetic efficiency, they differed in stress resilience. *B. subtilis* enhanced drought tolerance, as shown by increased relative water content, starch accumulation in chloroplasts, and elevated levels of l-tryptophan, previously linked to hydric stress responses [29]. Conversely, *B. velezensis* induced an intermediate phenotype under water-limited conditions, marked by some accumulation of l-tryptophan and increased levels of luteolin-7-glucoside [47]. Specialized metabolites with antifungal properties, including caffeic acid, rosmarinic acid, and a chlorogenic acid analog, were enriched in leaves of *Bacillus*-primed plants. Because the metabolite profiling relied on extensive within-plant sampling, the biological interpretation of the observed patterns has been supported with independent transcriptomic and physiological data. This metabolic reconfiguration likely contributes to enhanced resistance against *B. cinerea*, as observed in *B. subtilis*-treated plants. Notably, *B. velezensis* priming extended to plant–herbivore interactions. Seed treatment with *B. velezensis* induced an initial oxidative burst and downregulation of a key jasmonic acid biosynthesis gene (*AOS*), which may enhance the efficiency of JA-mediated defenses upon attack by spider mites, specifically those populations sensitive to this pathway (Fig. S7). Although infestation by the broadly adapted *T. urticae* strain Algarrobo-2 triggered strong transcriptional induction of JA biosynthetic genes, this response did not translate into reduced herbivore performance. In contrast, the JA-sensitive strain Santpoort-2 exhibited significantly reduced reproductive success on the same plants, despite a weaker induction of JA-associated transcripts. The differential performance of the two *T. urticae* strains tested likely reflects intraspecific variation in herbivore strategies for coping with plant defenses [48]. The Algarrobo-2 strain, known for its broad host range [37] and JA-resistance traits [38], maintained high performance on *Bacillus*-treated plants despite metabolomic shifts, possibly due to enhanced detoxification mechanisms [49] and the ability to degrade phenolic compounds [50]. By contrast, the Santpoort-2 strain, previously shown to be JA sensitive [38, 51], was more susceptible to defenses triggered by *B. velezensis* seed treatment, supporting the role of JA pathway priming in herbivore resistance. Importantly, the apparent decoupling between JA transcript abundance and resistance outcomes suggests that defensive effectiveness depends not solely on transcriptional magnitude, but on the timing, localization, or qualitative output of JA signaling. Such priming may facilitate rapid hormone activation, efficient conversion to bioactive jasmonates, or deployment of specific defensive metabolites that impair sensitive herbivores while remaining ineffective against broadly adapted strains with enhanced detoxification capacity. While these findings suggest potential interference with pest establishment, further studies under field-like conditions are needed to assess long-term population dynamics. Altogether, these findings indicate that early microbial priming can alter plant–herbivore interactions in a strain-specific manner, offering protective effects against key pests. Future work should explore the bidirectional influence between microbial colonization and herbivore-induced defenses, which may shape both microbiome composition and plant resistance traits over time [52, 53]. Certainly, although our work focused on host responses, the inoculation of seeds with *Bacillus* strains likely reshapes the seed and root microbiome. Early microbial colonizers are known to influence the assembly of microbial communities during plant development [54, 55]. Further studies will be required to assess how this restructuring affects long-term plant–microbiome dynamics and contributes to the phenotypes observed in this study.

In conclusion, our findings reveal that even among closely related mutualistic microbes, early interactions can lead to divergent physiological programs. These results challenge the assumption of functional redundancy among beneficial strains and highlight the central role of the host in modulating mutualistic outcomes, providing new insight into the diversification and plasticity of beneficial plant–microbe interactions.

## Materials and methods

### Microbial strains, plant material, and growth conditions

*B. subtilis* NCIB3610 and *B. velezensis* FZB42 (both from our laboratory collection) were cultured from cryopreserved stocks at 28°C on lysogeny broth (LB; 1% tryptone, 0.5% yeast extract, 0.5% NaCl) solid medium. The necrotrophic fungal pathogen *B. cinerea* isolate B05.10 was subcultured from frozen stocks prior to inoculation assays and maintained at 25°C on potato dextrose agar (PDA; Oxoid, Thermo Fisher Scientific, Waltham, MA, USA). Seeds of *C. melo* var. Rochet Panal (Semillas Fitó, Barcelona, Spain) were germinated on sterile, moistened filter paper placed in Petri dishes wrapped in aluminum foil and incubated in controlled-environment chambers (25°C, darkness) for 5 days to assess early plant responses. For long-term experiments assessing growth promotion and physiological parameters, seedlings were transplanted into pots and grown under controlled conditions (25°C, 16-h light/8-h dark photoperiod, 60% relative humidity, 300 μmol photons m^−2^ s^−1^). *A. thaliana* ecotype Columbia-0 (Col-0) seeds were surface sterilized, stratified in darkness for 48 h at 4°C, and germinated on Murashige and Skoog (MS) agar medium for radicle development analyses.

### Herbivores

Two *T. urticae* strains were used in this study. The red phenotype strain Algarrobo-2, originally isolated from crop fields in Málaga, Spain, was selected for its capacity to infest bean, melon, and tomato plants. The second strain, Santpoort-2, was kindly provided by Dr Merijn Kant (University of Amsterdam). This strain, derived from *Euonymus europaeus* in the Santpoort Nature Reserve, is characterized by its susceptibility to tomato-induced defenses [38, 56]. Mite colonies were maintained on detached *Phaseolus vulgaris* (cv. Canadian Wonder) leaves placed on moist cotton in climate-controlled chambers (25°C, 16-h light/8-h dark photoperiod). For bioassays, 16-day-old adult females were used. These were obtained by allowing adult females to oviposit on fresh bean leaves for 48 h; eggs were maintained for 16 days under identical environmental conditions before use.

### Quantitative analysis of radicle and plant growth

Seed inoculation with *Bacillus* spp. followed a previously described protocol [13]. Bacterial cultures were grown overnight at 28°C in liquid LB medium, harvested by centrifugation, washed twice in sterile distilled water, and resuspended to optical densities (OD_600_) corresponding to 10^7^ or 10^8^ CFU ml^−1^ for low- and high-dose treatments, respectively. Melon seeds were surface sterilized with 0.2% sodium hypochlorite and subsequently immersed in bacterial suspensions for 90 min at room temperature under gentle agitation. Water-treated seeds served as negative controls (mock treatment). Radicle length and area were quantified 5 days postinoculation using ImageJ software [57] (https://imagej.net/ij/). Measurements were normalized to the mean values of the mock treatment, which were set as 100%. Parallel treatments using purified bacterial metabolites like fengycin, surfactin, and bacillomycin D 20 μM (purification described below) were conducted following the same procedure. Plant fresh weight was recorded at designated time points and expressed relative to mock-treated controls.

### Bacterial colonization and quantification

For microbial load assessment, radicles were excised and placed in 1.5-ml microcentrifuge tubes containing 1 ml of phosphate-buffered saline (PBS) and ~ 1 g of sterile 3-mm glass beads. Samples were vortexed for 60 s to dislodge surface-associated bacteria. In parallel, whole seeds were homogenized in PBS using a sterile mortar and pestle. Serial dilutions of each suspension were plated on LB agar to determine total CFU per milliliter. To quantify endospores, aliquots of the same suspensions were heat treated at 80°C for 5 min prior to plating. For spatial analysis of bacterial colonization, antibiotic-resistant strains were used: *B. subtilis* (spectinomycin resistant) and *B. velezensis* (chloramphenicol resistant). Root tissues were homogenized in 10 ml of sterile water using a stomacher for 3 min, and serial dilutions were plated on selective media to determine total and spore-forming CFU counts.

### Hydric stress assay

Hydric stress tolerance was evaluated in 21-day-old plants derived from *Bacillus*-treated and control seeds. Control plants were irrigated twice weekly with 1.5 l of water per tray (16 plants). For drought assay, irrigation was withheld for 17 days and subsequently restored. Plant recovery was assessed 2 days after rewatering. Relative water content (RWC) was measured at three time points: before stress, during stress, and after rehydration. Six samples per treatment were collected, each consisting of four to five leaf discs (1.5-cm diameter). RWC was calculated as RWC (%) = (FW − DW) / (TW − DW) × 100, where FW is fresh weight, TW is turgid weight (after hydration at room temperature for 3–4 h), and DW is dry weight (after oven drying at 80°C for 24 h).

### Photosynthetic capacity analysis

Photosynthetic performance was assessed by variable chlorophyll fluorescence imaging (Chl-FI) using an Open FluorCam 700 MF system (Photon System Instruments, Brno, Czechia). Leaves were dark adapted for 30 min before imaging (Protocol 1 [58]). Minimum fluorescence (F_0_) was first recorded, followed by a saturating pulse (2500 μmol photons m^−2^ s^−1^) to determine maximum fluorescence (F_M_). Actinic light (400 μmol photons m^−2^ s^−1^) was then applied, and 11 successive saturating pulses were used to record Ft and F′_m_ at multiple time points (2–300 s). Fluorescence parameters were calculated using FluorCam software (v5.0) according to Ref. [59]: (a) maximum quantum yield of PSII [F_v_/F_M_ = (F_M_ − F_0_)/F_M_]; (b) effective quantum yield of PSII (Φ_PSII_ = (F′_M_ − F_0_)/F′_M_); and (c) nonphotochemical quenching [NPQ = (F_M_ − F′_M_)/F′_M_]. False-color images were generated to visualize the spatial distribution of these parameters.

### Quantification of chlorophyll *a* and chlorophyll *b*

Chlorophyll quantification was performed according to Ref. [60]. Three leaf discs (1.5-cm diameter) per plant were collected from 10 plants per treatment group. Pigments were in 80% acetone, followed by centrifugation at 9000 × *g* for 5 min. Absorbance of the supernatant was measured at 647 and 663 nm using a spectrophotometer. Chlorophyll concentrations were calculated as follows:

[Chl a] (μg ml^−1^) = 12.25*A*_663_ − 2.79A_647_ and [Chl b] (μg ml^−1^) = 21.5*A*_647_ − 5.10*A*_663_ and expressed as microgram per centimeter squared of leaf tissue.

### Thermal imaging

Infrared (IR) images of leaves from mock-treated and *Bacillus*-treated plants, under both uninfected and *B. cinerea*-infected conditions, were captured mid-morning using a Photon A305sc camera (FLIR Systems, Wilsonville, OR, USA) vertically positioned above the attached leaves, following Ref. [61]. The camera provides images at a resolution of 320 × 240 pixels and a thermal sensitivity <0.05°C within the spectral range of 7.5–13 μm. Temperature data were extracted and analyzed using FLIR R&D software v3.4. Eleven regions of interest (ROI) per plant were evaluated. Representative false-color thermal images are shown in the figures.

### BGF imaging

Phenolic compounds in plant tissues emit blue (F440) and green (F520) fluorescence when excited under UV light. These signals were recorded using a customized Open FluorCam FC 800-O system (Photon Systems Instruments, Brno, Czechia), following the procedure described in Ref. [62]. Leaves were positioned beneath the camera and illuminated at 355 nm. For each fluorescence channel, nine frames were acquired with an exposure time of 800 ms using the appropriate cut-off filters. Frames were averaged to generate grayscale images (resolution: 740 × 480 pixels) representing F440 and F520 emissions. Imaging was conducted on leaves from mock-treated and *Bacillus*-treated plants, under both uninfected and *B. cinerea*-infected conditions. Image acquisition, data extraction, and false-color rendering were performed using FluorCam software version 7.1.0.3.

### Transmission electron microscopy

Leaf sections (~0.5 × 0.5 cm) were fixed in 4% paraformaldehyde and 2.5% glutaraldehyde in 0.1 M phosphate buffer (PB) overnight at 4°C. Samples were washed in PB, postfixed with 1% osmium tetroxide for 90 min at room temperature, and dehydrated through a graded ethanol series (30%–100%). In-block staining was performed with 2% uranyl acetate in 50% ethanol overnight at 4°C. Samples were infiltrated sequentially with LR White resin/ethanol (1:1 and 3:1, 4 h each), followed by pure resin overnight, and polymerized at 70°C for 72 h. Ultrathin sections were observed using a FEI TALOS F200X transmission electron microscope (Thermo Fisher Scientific). Starch granule area (μm^2^) was quantified using ImageJ from at least five regions per section, across three biological replicates per treatment.

### Scanning electron microscopy

Leaf samples were fixed in 2.5% glutaraldehyde in 0.1 M PB overnight at 4°C. After washing in PB, samples were dehydrated through a graded ethanol series (50%, 60%, 70%, 80%, 90%, and 100% twice) and subjected to critical point drying. The total number of stomata was counted from at least five independent regions per sample, across three biological replicates per treatment. Stomatal density (S mm^−2^) was calculated by dividing the total number of stomata by the corresponding leaf area.

### RNA sequencing and transcriptomic analysis

Total RNA was extracted from biological duplicates using the RNAprep Pure Tissue Kit (TIANGEN, China). Library construction and sequencing were performed by Novogene (Beijing, China) on Illumina NovaSeq 6000 and NovaSeqX Plus platforms. For long noncoding RNA (lncRNA) profiling, ribosomal RNA (rRNA) was removed using the TruSeq Stranded Total RNA Library Prep Kit, followed by ethanol precipitation to eliminate residual rRNA. rRNA-depleted RNA was used to construct libraries with the NEBNext® Ultra™ Directional RNA Library Prep Kit (New England Biolabs), involving RNA fragmentation, first-strand synthesis using random hexamer primers, second-strand synthesis with dUTP substitution for dTTP, and subsequent end repair, A-tailing, adaptor ligation, size selection, USER enzyme treatment, PCR amplification, and purification steps. For small RNA libraries, 3′ and 5′ adaptors were ligated to RNA ends, followed by reverse transcription, PCR enrichment, and purification. Libraries with insert sizes of 18–40 bp were selected for sequencing (single-end 50-bp reads, SE50). Library quality and concentration were evaluated using a Qubit 3.0 fluorometer, qPCR, and an Agilent 2100 Bioanalyzer. Sequencing of lncRNA, mRNA, and other ncRNA libraries was performed with paired-end 150-bp reads (PE150) on the NovaSeqX Plus platform, generating ~12 Gb (~40 million reads per sample). Small RNA libraries were sequenced on the NovaSeq6000 platform (SE50, ~10 million reads per sample). Differential gene expression analysis was conducted using Novogene bioinformatic pipelines, aligning clean reads to the *C. melo* cv. DHL92 v4 reference genome [63].

### Metabolite extraction from plant tissues

Adult melon plants derived from *Bacillus*-treated and control seeds were sectioned to analyze the whole-plant metabolome. Roots, stems, and leaves were divided into multiple segments (three to four sections from each leaf). Metabolites were extracted by adding 1 ml of 80% HPLC-grade methanol to each sample, followed by vigorous vortexing. Due to the large number of sections analyzed per plant, only one biological replicate was processed. Tissue mass normalization was not feasible; however, the size of each plant section was kept constant. To enable relative quantitative comparisons, metabolite peak areas were normalized to total ion count, a widely accepted approach in nontargeted metabolomics [64]. Sulfamethazine (1 μM) was added to all samples as an internal standard and used for normalization during data analysis. Following a 2-h incubation, extracts were centrifuged at 14 000 × *g* for 30 min. Supernatants were collected and stored at −80°C until liquid chromatography–tandem mass spectrometry (LC–MS/MS) analysis. Extraction controls were prepared by processing three blank samples containing only pure methanol. Metabolite abundance was estimated based on relative comparisons among equally treated samples, as authentic standards were not available in the nontargeted metabolomics approach.

### Liquid chromatography–tandem mass spectrometry

Nontargeted metabolomics analyses were performed using a Q-Exactive Quadrupole-Orbitrap mass spectrometer coupled to a Vanquish ultra-high-performance liquid chromatography (UHPLC) system (Thermo Fisher Scientific), following the protocol described in Ref. [65]. Aliquots of 5 μl from each extract were injected into a UHPLC system equipped with a Kinetex C18 core-shell column (50 × 2 mm, 1.8-μm particle size, 100 Å pore size; Phenomenex, Torrance, USA). Chromatographic separation was carried out at a flow rate of 0.5 ml min^−1^ using solvent A (H₂O with 0.1% formic acid) and solvent B (acetonitrile with 0.1% formic acid) as the mobile phases. The linear gradient was programmed as follows: 0–0.5 min, 5% B; 0.5–4 min, 5%–50% B; 4–5 min, 50%–99% B; 5–7 min, washout at 99% B; and 7–9 min, 5% B (re-equilibration). Electrospray ionization (ESI) was operated in positive-ion mode under the following settings: sheath gas flow at 35 AU, auxiliary gas flow at 10 AU, sweep gas flow at 2 AU, auxiliary gas temperature at 400°C, spray voltage at 3.5 kV, capillary temperature at 250°C, and S-lens voltage at 50 V. MS/MS spectra were acquired in data-dependent acquisition (DDA) mode. Full MS1 scans (*m/z* 150–1500) were recorded at a resolution of 17 500, followed by up to five MS/MS scans per duty cycle. The C-trap fill time was limited to a maximum of 100 ms or until the automatic gain control (AGC) target of 5 × 10^5^ ions was reached. Precursor ions were isolated using a 1 *m/z* quadrupole window and fragmented with normalized collision energies (NCE) of 20%, 30%, and 40%, assuming a default charge state of *z* = 1. Apex triggering was applied to acquire MS/MS scans 2–15 s after precursor detection in the survey scan. A dynamic exclusion window of 5 seconds was used. Precursor ions with unassigned charge states and isotope peaks were excluded from fragmentation.

### Feature-based molecular networking and spectral library search

Following LC–MS/MS data acquisition, MS1 and MS/MS feature extraction was performed using MZmine software version 3.9.0 [66]. Peak detection thresholds were set to 1 × 10^5^ for MS1 and 1 × 10^3^ for MS/MS. For chromatogram building, a mass accuracy of 10 ppm and a minimum peak intensity of 5 × 10^5^ were applied. Extracted ion chromatograms (XICs) were deconvoluted using the baseline cutoff algorithm with a minimum intensity threshold of 1 × 10^5^. After deconvolution, XICs were matched to MS/MS spectra using a 0.02 *m/z* and 0.2-min retention time window. Isotopic peaks were grouped, and features across samples were aligned with a mass tolerance of 10 ppm and a retention time tolerance of 0.1 min. MS1 features lacking corresponding MS/MS spectra, isotopic peaks, or present in fewer than three samples were removed. Gaps in the feature matrix were filled using a relaxed retention time tolerance of 0.2 min and a mass tolerance of 10 ppm. The final feature table was exported in .csv format, and the corresponding MS/MS spectra were saved as .mgf files. Contaminant features detected in blanks were filtered out; only those with a blank-to-average abundance ratio below 30% were retained. Feature-based molecular networking (FBMN) and spectral library matching were conducted on the GNPS platform (https://gnps.ucsd.edu/ProteoSAFe/static/gnps-splash.jsp) [67]. Networking parameters included a minimum cosine score of 0.7, precursor ion mass tolerance of 0.01 Da, fragment ion mass tolerance of 0.01 Da, a minimum of four matched fragment peaks, and a minimum cluster size of one (MS-Cluster deactivated). For analog searches, the same cosine threshold (0.7) was used with a maximum allowed mass difference of 100 *m/z*. Resulting molecular networks were visualized in Cytoscape version 3.10.2 [68]. Chemical structure annotations were enriched through *in silico* predictions using SIRIUS 4 software (https://bio.informatik.uni-jena.de/software/sirius/) [69], comparing results against internal databases including Bio Database, GNPS, Natural Products, PubChem, and PubMed. Chemical classes were annotated using NPClassifier [70] and ClassyFire [71] ontologies. Mirror plots comparing *mzspec* values of selected features with database records were generated using the USI resolver tool (https://metabolomics-usi.ucsd.edu/) (Fig. S8). Annotations followed the guidelines described in Ref. [30] (Tables S2 and S3). Statistical analyses were performed in Metaboanalyst v.5.0 [72] (https://www.metaboanalyst.ca/) using the quantitative table filtered by interquartile range (IQR) and normalized to the internal standard sulfamethazine.

### 3D mass spectral molecular cartography and visualization

Collection of plant material for 3D mass spectral molecular cartography and 3D modeling was done as described in Ref. [13]. Point coordinates and LC–MS data were combined into a .csv file and mapped on 3D models using Ili software [73] (https://ili.ucsd.edu/).

### Lipopeptide extraction and purification

Lipopeptides were extracted from *B. subtilis* 3610 and *B. velezensis* FZB42 cultures grown in liquid medium for 48 h. After incubation, cultures were centrifuged (25 min, 8500 × *g*, 4°C), and the supernatant was collected. Lipopeptides were precipitated by acidification to pH 2 with 6 N HCl and incubated overnight at 4°C. The resulting precipitate was collected by centrifugation (25 min, 8500 × *g*, 4°C) and extracted with methanol under continuous agitation overnight. Insoluble residues were removed by centrifugation, and the supernatant was concentrated by rotary evaporation to obtain crude lipopeptide extracts. Purification was performed by solid-phase extraction (SPE) using Strata C18-U cartridges (200 mg, 3 ml column volume; Phenomenex, Torrance, CA, USA). Columns were preactivated with one column volume (CV) of methanol and equilibrated with one CV of Milli-Q water. Samples were loaded and sequentially eluted with Milli-Q water followed by increasing concentrations of methanol in water (25%–100%, 1 CV each). Eluted fractions were analyzed by thin-layer chromatography (TLC) and high-performance liquid chromatography (HPLC). TLC was conducted on silica gel plates using a dichloromethane/methanol/water (65:25:4) mobile phase. Plates were air-dried and sprayed with water to visualize lipopeptides. HPLC analyses were carried out on an Agilent 1260 Infinity II system (Agilent Technologies, Santa Clara, CA, USA) with UV detection at 210 nm. The analytical phase used a Zorbax Eclipse Plus C18 column (250 × 4.6 mm, 5 μm; Agilent), with 20-μl injections of each SPE fraction, while the preparative phase employed a Zorbax Eclipse Plus C18 column (250 × 9.8 mm, 5 μm; Agilent) with 350-μl injections of the 75% and 100% methanol SPE fractions. Elution was performed using acetonitrile and 0.1% trifluoroacetic acid in Milli-Q water under gradient conditions. Lipopeptides were collected according to retention times: 40/60 (bacillomycin D), 50/50 (fengycin), and 80/20 (surfactin), and concentrated under a nitrogen stream. Lipopeptide identity was confirmed by MALDI-TOF mass spectrometry (UltrafleXtreme, Bruker, Billerica, MA, USA). Detected *m/z* values were compared against reference spectra for bacillomycin D, fengycin, and surfactin.

### *In vitro* antifungal activity assays

Commercial compounds were tested for inhibitory activity against *B. cinerea* spores and mycelia. Spore germination inhibition was evaluated in 96-well plates (final volume, 100 μl), containing 100 spores per well. Absorbance was measured at 600 nm after 48 h of incubation. For mycelial inhibition, 1 ml of saturated potato dextrose broth (PDB) containing fungal mycelium was added to each well. After 24 h, mycelial fresh weight was determined following filtration through 0.45-μm filters. PDB containing ethanol (at the maximum concentration used for compound solubilization) served as the control.

### Bacterial growth and biofilm formation

Cytotoxicity of commercial compounds against *B. subtilis* and *B. velezensis* was evaluated by monitoring bacterial growth and biofilm formation. For growth assays, commercial compounds were added to 5 ml of LB medium inoculated with 10 μl of overnight bacterial culture. Total = CFU per milliliter was determined as described above. Biofilm formation was assessed in Msgg medium (100 mM morpholinepropane sulfonic acid, pH 7; 0.5% glycerol; 0.5% glutamate; 5 mM potassium phosphate, pH 7; 50 μg ml^−1^ tryptophan; 50 μg ml^−1^ phenylalanine; 50 μg ml^−1^ threonine; 2 mM MgCl_2_; 700 μM CaCl_2_; 50 μM FeCl_3_; 50 μM MnCl_2_; 2 μM thiamine; 1 μM ZnCl_2_) using 48-well plates. Washed cultures (10 μl) were added to 1-ml medium and incubated at 28°C. Biofilm development was evaluated after 24 h of incubation.

### Fungal infection assays

*Botrytis cinerea* assays were performed on 3–4-week-old plants derived from *Bacillus*-treated or control seeds. Conidial suspensions were prepared in sterile water, filtered through 40-μm mesh, and adjusted to 10^5^ spores ml^−1^ in 0.5× filtered organic grape juice. Two 5-μl droplets of inoculum were applied to the second leaf of each plant. Plants were placed in trays containing a shallow layer of water and sealed with transparent plastic domes and parafilm to maintain high humidity. Lesion development was monitored at 1, 3, and 8 days postinoculation (dpi). Lesion area was quantified using ImageJ software and normalized to mock-infected controls (set as 100%).

### Performance assays of *T. urticae*

Reproductive performance of *T. urticae* Algarrobo-2 and Santpoort-2 strains was assessed as described in Ref. [38]. Briefly, five adult females were transferred to 2- to 3-cm leaf arenas bordered with lanolin (Sigma-Aldrich). Three arenas (two on the first leaf and one on the second) were established per plant, using four plants per treatment. After 4 days, eggs were counted under a stereomicroscope and reproductive performance was calculated for each arena as follows: No. of eggs/[live mites + 0.5(total mites − live mites)].

### Induction of plant defense responses

Induction of plant defenses was evaluated by measuring the expression of defense-related marker genes. Leaf sections (2- to 3-cm diameter, bordered with lanolin) were infested with 15 adult female mites (three sections per plant, six plants per treatment). To determine the basal expression of defense genes, six plants per treatment were left uninfested. At 4 days postinfestation (dpi), both infested and noninfested leaf sections were excised, flash frozen in liquid nitrogen, and stored at −80°C. Tissue from three sections per plant was pooled as one biological replicate. To evaluate defense responses under different physiological states, plants from each treatment were analyzed under two conditions: noninfested (representing the basal defense state induced by *Bacillus* priming) and mite infested (representing the induced defense response by *T. urticae* strains). Total RNA was extracted using the NucleoSpin RNA Plant kit (Macherey-Nagel, Fisher Scientific), and 2 μg of DNase-treated RNA was used for cDNA synthesis. Quantitative reverse transcription PCR (RT-PCR) was performed with 1.5 μl of 5× diluted cDNA and SsoAdvanced Universal SYBR Green Supermix (Bio-Rad) on a CFX Opus 384 Real-Time PCR System (Bio-Rad). Expressions of *PR1-1a*, *AOC*, and *AOS* (primer sequences in Table S4) were analyzed. Primer efficiency was validated as in Ref. [74]. Gene expression was normalized to *C. melo Actin-7* and calculated using the ΔΔCt method [75]. Data were expressed as fold changes relative to mock-treated noninfested control plants, while all statistical analyses were performed on normalized ΔCt data. Each assay used six biological replicates and was performed in triplicate.

### Statistical analysis

Statistical analyses were performed using GraphPad Prism version 9 (GraphPad Software, Boston, MA, USA; www.graphpad.com). The specific tests used are indicated in the corresponding figure legends. *P* values <0.05 were considered significant. Asterisks denote significance levels as follows: ^*^P < 0.05, ^**^P < 0.01, ^***^P < 0.001, ^****^P < 0.0001. Metabolomics data were analyzed using Metaboanalyst v.5.0 [72] (https://www.metaboanalyst.ca) after interquartile range (IQR) filtering and normalization to the internal standard, sulfamethazine. All experiments were independently replicated at least three times with comparable results.

## Supplementary Material

Web_Material_uhag053

## Data Availability

All the raw RNA-seq data have been submitted to the Gene Expression Omnibus (GEO) and can be accessed through GEO series accession GSE299630. Metabolomics data are deposited at https://massive.ucsd.edu/ with identifier MSV000098118. All data are available within this article and its supporting information.

## References

[1] Martin FM, Uroz S, Barker DG. Ancestral alliances: plant mutualistic symbioses with fungi and bacteria. Science. 2017;356:10.1126/science.aad4501 28546156

[2] Soto MJ, Domínguez-Ferreras A, Pérez-Mendoza D. et al. Mutualism versus pathogenesis: the give-and-take in plant-bacteria interactions. Cell Microbiol. 2009;11:381–8 19134114 10.1111/j.1462-5822.2009.01282.x

[3] Mesny F, Hacquard S, Thomma BP. Co-evolution within the plant holobiont drives host performance. EMBO Rep. 2023;24:EMBR202357455 10.15252/embr.202357455 PMC10481671 37471099

[4] Berg G, Raaijmakers JM. Saving seed microbiomes. ISME J. 2018;12:1167–70 29335636 10.1038/s41396-017-0028-2 PMC5931960

[5] Shade A, Jacques MA, Barret M. Ecological patterns of seed microbiome diversity, transmission, and assembly. Curr Opin Microbiol. 2017;37:15–22 28437661 10.1016/j.mib.2017.03.010

[6] Abdelfattah A, Tack AJM, Lobato C. et al. From seed to seed: the role of microbial inheritance in the assembly of the plant microbiome. Trends Microbiol. 2023;31:346–55 36481186 10.1016/j.tim.2022.10.009

[7] Tsotetsi T, Nephali L, Malebe M. et al. Bacillus for plant growth promotion and stress resilience: what have we learned? Plants. 2022;11:2482 10.3390/plants11192482 PMC9571655 36235347

[8] Blake C, Christensen MN, Kovacs AT. Molecular aspects of plant growth promotion and protection by *Bacillus subtilis*. Mol Plant Microbe Interact. 2021;34:15–25 32986513 10.1094/MPMI-08-20-0225-CR

[9] Berendsen RL. et al. Disease-induced assemblage of a plant-beneficial bacterial consortium. ISME J. 2018;12:1496–507 29520025 10.1038/s41396-018-0093-1 PMC5956071

[10] Abd El-Daim IA, Bejai S, Meijer J. *Bacillus velezensis* 5113 induced metabolic and molecular reprogramming during abiotic stress tolerance in wheat. Sci Rep. 2019;9:16282 10.1038/s41598-019-52567-x PMC6841942 31704956

[11] Ma KW. et al. Coordination of microbe–host homeostasis by crosstalk with plant innate immunity. Nat Plants. 2021;7:814–25 34031541 10.1038/s41477-021-00920-2 PMC8208891

[12] Laurich JR, Lash E, O’Brien AM. et al. Community interactions among microbes give rise to host-microbiome mutualisms in an aquatic plant. MBio. 2024;15:e00972-24 10.1128/mbio.00972-24 PMC11324027 38904411

[13] Berlanga-Clavero MV. et al. *Bacillus subtilis* biofilm matrix components target seed oil bodies to promote growth and anti-fungal resistance in melon. Nat Microbiol. 2022;7:1001–15 35668112 10.1038/s41564-022-01134-8 PMC9246715

[14] Gu Q, Yang Y, Yuan Q. et al. Bacillomycin D produced by *Bacillus amyloliquefaciens* is involved in the antagonistic interaction with the plant pathogenic fungus *Fusarium graminearum*. Appl Environ Microbiol. 2017;83:e01075-17 10.1128/AEM.01075-17 PMC5601353 28733288

[15] Roquis D. et al. Genomic impact of stress-induced transposable element mobility in *Arabidopsis*. Nucleic Acids Res. 2021;49:10431–47 34551439 10.1093/nar/gkab828 PMC8501995

[16] Ruan J, Zhou Y, Zhou M. et al. Jasmonic acid signaling pathway in plants. Int J Mol Sci. 2019;20:2479 10.3390/ijms20102479 PMC6566436 31137463

[17] Howe GA, Jander G. Plant immunity to insect herbivores. Annu Rev Plant Biol. 2008;59:41–66 18031220 10.1146/annurev.arplant.59.032607.092825

[18] Di Ferdinando M, Brunetti C, Fini A. et al. Flavonoids as antioxidants in plants under abiotic stresses. In: Ahmad, P., Prasad, M. (eds) Abiotic Stress Responses in Plants. New York, NY: Springer, 2012, 159–79

[19] Evans JR . Improving photosynthesis. Plant Physiol. 2013;162:1780–93 23812345 10.1104/pp.113.219006 PMC3729760

[20] Maxwell K, Johnson GN. Chlorophyll fluorescence-a practical guide. J Exp Bot. 2000;51:659–68 10938857 10.1093/jxb/51.345.659

[21] Foyer CH, Shigeoka S. Understanding oxidative stress and antioxidant functions to enhance photosynthesis. Plant Physiol. 2011;155:93–100 21045124 10.1104/pp.110.166181 PMC3075779

[22] Lawlor DW, Tezara W. Causes of decreased photosynthetic rate and metabolic capacity in water-deficient leaf cells: a critical evaluation of mechanisms and integration of processes. Ann Bot. 2009;103:561–79 19155221 10.1093/aob/mcn244 PMC2707350

[23] Graf A, Schlereth A, Stitt M. et al. Circadian control of carbohydrate availability for growth in *Arabidopsis* plants at night. Proc Natl Acad Sci U S A. 2010;107:9458–63 20439704 10.1073/pnas.0914299107 PMC2889127

[24] Wu L, Birch RG. Isomaltulose is actively metabolized in plant cells. Plant Physiol. 2011;157:2094–101 22010106 10.1104/pp.111.189001 PMC3327191

[25] Fernie AR, Roessner U, Geigenberger P. The sucrose analog palatinose leads to a stimulation of sucrose degradation and starch synthesis when supplied to discs of growing potato tubers 1. Plant Physiol. 2001;125:1967–77 11299376 10.1104/pp.125.4.1967 PMC88852

[26] Thalmann M. et al. Regulation of leaf starch degradation by abscisic acid is important for osmotic stress tolerance in plants. Plant Cell. 2016;28:1860–78 27436713 10.1105/tpc.16.00143 PMC5006701

[27] Kibler CL, Trugman AT, Roberts DA. et al. Evapotranspiration regulates leaf temperature and respiration in dryland vegetation. Agric For Meteorol. 2023;339:109560

[28] Hubbart S, Smillie IRA, Heatley M. et al. Enhanced thylakoid photoprotection can increase yield and canopy radiation use efficiency in rice. Commun Biol. 2018;1:22 10.1038/s42003-018-0026-6 PMC6123638 30271909

[29] Sadak MS, Ramadan AAEM. Impact of melatonin and tryptophan on water stress tolerance in white lupine (*Lupinus termis* L.). Physiol Mol Biol Plants. 2021;27:469–81 33854277 10.1007/s12298-021-00958-8 PMC7981349

[30] Sumner LW. et al. Proposed minimum reporting standards for chemical analysis: chemical analysis working group (CAWG) metabolomics standards initiative (MSI). Metabolomics. 2007;3:211–21 24039616 10.1007/s11306-007-0082-2 PMC3772505

[31] Barón M, Moreno-Martín MT, Pineda M. Uncovering *Botrytis cinerea*-induced physiological changes in melon plants using multi-sensor imaging approaches. Plant Stress. 2025;15:100769

[32] Gitelson AA, Buschmann C, Lichtenthaler HK. Leaf chlorophyll fluorescence corrected for re-absorption by means of absorption and reflectance measurements. J Plant Physiol. 1998;152:283–96

[33] Erb M, Kliebenstein DJ. Plant secondary metabolites as defenses, regulators, and primary metabolites: the blurred functional trichotomy. Plant Physiol. 2020;184:39–52 32636341 10.1104/pp.20.00433 PMC7479915

[34] Kant MR, Bleeker PM, Van Wijk M. et al. Plant volatiles in defence. Adv Bot Res. 2009;51:613–66

[35] Wink M, Schmeller T, Latz-Brüning B. Modes of action of allelochemical alkaloids: interaction with neuroreceptors, DNA, and other molecular targets. J Chem Ecol. 1998;24:1881–937

[36] Li B, Liu F, He X. et al. Leaf beetle symbiotic bacteria degrade chlorogenic acid of poplar induced by egg deposition to enhance larval survival. Plant Cell Environ. 2025;48:4212–26 39925102 10.1111/pce.15427

[37] Bolland HR, Gutierrez J, Flechtmann Brill CHW. et al. World Catalogue of the Spider Mite Family. Acari: BRILL; 1998:

[38] Alba JM. et al. Spider mites suppress tomato defenses downstream of jasmonate and salicylate independently of hormonal crosstalk. New Phytol. 2015;205:828–40 25297722 10.1111/nph.13075 PMC4301184

[39] Han Z, Xiong D, Schneiter R. et al. The function of plant PR1 and other members of the CAP protein superfamily in plant–pathogen interactions. Mol Plant Pathol. 2023;24:651–68 36932700 10.1111/mpp.13320 PMC10189770

[40] Stenzel I. et al. Jasmonate biosynthesis and the allene oxide cyclase family of *Arabidopsis thaliana*. Plant Mol Biol. 2003;51:895–911 12777050 10.1023/a:1023049319723

[41] Beyer SF, Bel PS, Flors V. et al. Disclosure of salicylic acid and jasmonic acid-responsive genes provides a molecular tool for deciphering stress responses in soybean. Sci Rep. 2021;11:20600 10.1038/s41598-021-00209-6 PMC8523552 34663865

[42] Grifé-Ruiz M, Hierrezuelo-León J, de Vicente A. et al. Diversification of lipopeptide analogues drives versatility in biological activities. J Agric Food Chem. 2025;73:1403–16 39760433 10.1021/acs.jafc.4c11372 PMC11741111

[43] Crisp PA, Ganguly D, Eichten SR. et al. Reconsidering plant memory: intersections between stress recovery, RNA turnover, and epigenetics. Sci Adv. 2016;2:e1501340 10.1126/sciadv.1501340 PMC4788475 26989783

[44] Wu W, Fan G. The role of epigenetics in plant pathogens interactions under the changing environments: a systematic review. Plant Stress. 2025;15:100753

[45] Hannan Parker A, Wilkinson SW, Ton J. Epigenetics: a catalyst of plant immunity against pathogens. New Phytol. 2022;233:66–83 34455592 10.1111/nph.17699

[46] Mierziak J, Wojtasik W. Epigenetic weapons of plants against fungal pathogens. BMC Plant Biol. 2024;24:175 10.1186/s12870-024-04829-8 PMC10916060 38443788

[47] Mechri B, Tekaya M, Hammami M. et al. Effects of drought stress on phenolic accumulation in greenhouse-grown olive trees (*Olea europaea*). Biochem Syst Ecol. 2020;92:104112

[48] Kant MR. et al. Mechanisms and ecological consequences of plant defence induction and suppression in herbivore communities. Ann Bot. 2015;115:1015–51 26019168 10.1093/aob/mcv054 PMC4648464

[49] Grbić M. et al. The genome of *Tetranychus urticae* reveals herbivorous pest adaptations. Nature. 2011;479:487–92 22113690 10.1038/nature10640 PMC4856440

[50] Njiru C, Xue W, de Rouck S. et al. Intradiol ring cleavage dioxygenases from herbivorous spider mites as a new detoxification enzyme family in animals. BMC Biol. 2022;20:131 10.1186/s12915-022-01323-1 PMC9167512 35658860

[51] Chafi R. et al. Competitor displacement by an herbivore that manipulates plant defences. Preprint at 2024. 10.1101/2024.05.20.594407

[52] Singh DP, Maurya S, Satnami L. et al. Roots of resistance: unraveling microbiome-driven plant immunity. Plant Stress. 2024;14:100661

[53] Pirttilä AM, Brusila V, Koskimäki JJ. et al. Exchange of microbiomes in plant-insect herbivore interactions. MBio. 2023;14:e03210-22 10.1128/mbio.03210-22 PMC10127587 36880763

[54] Wang X, Li Y, Rensing C. et al. Early inoculation and bacterial community assembly in plants: a review. Microbiol Res. 2025;296:128141 40120566 10.1016/j.micres.2025.128141

[55] Hadian S, Smith DL, Supronienė S. Modulating the plant microbiome: effects of seed inoculation with endophytic bacteria on microbial diversity and growth enhancement in pea plants. Microorganisms. 2025;13:570 10.3390/microorganisms13030570 PMC11945133 40142462

[56] Kant MR, Sabelis MW, Haring MA. et al. Intraspecific variation in a generalist herbivore accounts for differential induction and impact of host plant defences. Proc R Soc B Biol Sci. 2008;275:443–52 10.1098/rspb.2007.1277 PMC2596823 18055390

[57] Schneider CA, Rasband WS, Eliceiri KW. NIH image to ImageJ: 25 years of image analysis. Nat Methods. 2012;9:671–5 22930834 10.1038/nmeth.2089 PMC5554542

[58] Pineda M, Soukupová J, Matouš K. et al. Conventional and combinatorial chlorophyll fluorescence imaging of tobamovirus-infected plants. Photosynthetica. 2008;46:441–51

[59] Polonio Á, Pineda M, Bautista R. et al. RNA-seq analysis and fluorescence imaging of melon powdery mildew disease reveal an orchestrated reprogramming of host physiology. Sci Rep. 2019;9:7978 10.1038/s41598-019-44443-5 PMC6538759 31138852

[60] Lichtenthaler HK, Buschmann C. Chlorophylls and carotenoids: measurement and characterization by UV-VIS spectroscopy. Curr Protocol Food Anal Chem. 2001;1:1–8

[61] Pineda M, Pérez-Bueno ML, Barón M. Novel vegetation indices to identify broccoli plants infected with *Xanthomonas campestris* pv. *campestris*. Front Plant Sci. 2022;13:790268 10.3389/fpls.2022.790268 PMC9265216 35812917

[62] Pérez-Bueno ML, Pineda M, Díaz-Casado E. et al. Spatial and temporal dynamics of primary and secondary metabolism in *Phaseolus vulgaris* challenged by pseudomonas syringae. Physiol Plant. 2015;153:161–74 24871330 10.1111/ppl.12237

[63] Ruggieri V, Alexiou KG, Morata J. et al. An improved assembly and annotation of the melon (*Cucumis melo* L.) reference genome. Sci Rep. 2018;8:8088 10.1038/s41598-018-26416-2 PMC5967340 29795526

[64] Sysi-Aho M, Katajamaa M, Yetukuri L. et al. Normalization method for metabolomics data using optimal selection of multiple internal standards. BMC Bioinform. 2007;8:93 10.1186/1471-2105-8-93 PMC1838434 17362505

[65] Petras D. et al. Mass spectrometry-based visualization of molecules associated with human habitats. Anal Chem. 2016;88:10775–84 27732780 10.1021/acs.analchem.6b03456 PMC6326777

[66] Schmid R. et al. Integrative analysis of multimodal mass spectrometry data in MZmine 3. Nat Biotechnol. 2023;41:447–9 36859716 10.1038/s41587-023-01690-2 PMC10496610

[67] Wang M. et al. Sharing and community curation of mass spectrometry data with global natural products social molecular networking. Nat Biotechnol. 2016;34:828–37 27504778 10.1038/nbt.3597 PMC5321674

[68] Shannon P. et al. Cytoscape: a software environment for integrated models of biomolecular interaction networks. Genome Res. 2003;13:2498–504 14597658 10.1101/gr.1239303 PMC403769

[69] Dührkop K. et al. SIRIUS 4: a rapid tool for turning tandem mass spectra into metabolite structure information. Nat Methods. 2019;16:299–302 30886413 10.1038/s41592-019-0344-8

[70] Kim HW. et al. NPClassifier: a deep neural network-based structural classification tool for natural products. J Nat Prod. 2021;84:2795–807 34662515 10.1021/acs.jnatprod.1c00399 PMC8631337

[71] Djoumbou Feunang Y. et al. ClassyFire: automated chemical classification with a comprehensive, computable taxonomy. J Chem. 2016;8:1–20 10.1186/s13321-016-0174-y PMC5096306 27867422

[72] Pang Z, Xu L, Viau C. et al. MetaboAnalystR 4.0: a unified LC-MS workflow for global metabolomics. Nat Commun. 2024;15:3675 10.1038/s41467-024-48009-6 PMC11063062 38693118

[73] Protsyuk I. et al. 3D molecular cartography using LC-MS facilitated by Optimus and ‘ili software. Nat Protoc. 2018;13:134–54 29266099 10.1038/nprot.2017.122

[74] Michán C, Pueyo C. Growth phase-dependent variations in transcript profiles for thioredoxin- and glutathione-dependent redox systems followed by budding and hyphal *Candida albicans* cultures. FEMS Yeast Res. 2009;9:1078–90 19702871 10.1111/j.1567-1364.2009.00558.x

[75] Livak KJ, Schmittgen TD. Analysis of relative gene expression data using real-time quantitative PCR and the 2-ΔΔCT method. Methods. 2001;25:402–8 11846609 10.1006/meth.2001.1262

