# Supplementary material for: Early seed priming with closely related Bacillus strains induces divergent physiological and defense responses in melon
Source: Hortic Res. 2026 Feb 23;13(6):uhag053. doi: 10.1093/hr/uhag053 (PMC13241187; doi:10.1093/hr/uhag053)
Supplement: Web_Material_uhag053 [file web_material_uhag053.zip › Supplementary Tables_major_revision.pdf]

Table S1. Differential expression (log_2 fold change) of selected genes in seeds and radicles treated with low and high doses of *B. velezensis*. Ordered by gene ID.

			Seed		Radicle	
**ID**	**Description**	**Abbr.**	**Low**	**High**	**Low**	**High**
MELO3C002457	Peroxidase	Perox.	-	-1.605	-	-1.048
MELO3C005540	14 kDa proline-rich protein DC2.15-like	PRP	-	-1.488	-	-
MELO3C008512	Pentatricopeptide repeat-containing protein	PPR	-	-	-9.367	1.917
MELO3C009108	Non-specific lipid-transfer protein	NSLTP	-1.168	-1.593	-1.218	-
MELO3C011383	DNA-directed RNA polymerase subunit beta	RNApol subB	-4.651	-3.119	2.918	-
MELO3C014217	HTH myb-type domain-containing protein	HTH	-	2.26	1.318	-
MELO3C014630	Lipoxygenase	LOX	-	-	-2.981	2.46
MELO3C018413	Allene oxide synthase	AOS	-1.717	-2.891	-	-
MELO3C020689	Respiratory burst oxidase	RBOH	-	-	-	7.002
MELO3C027069	Proteasome subunit beta	Prot.	-	-13.095	14.356	13.849
MELO3C027194	DNA-directed RNA polymerase II subunit RPB7	RNApol subR	-	-10.823	10.766	10.222
MELO3C027784	Gag/pol protein	Gag/pol	-	9.693	-	-
MELO3C028181	Gag/pol protein	Gag/pol	-	9.242	-	-
MELO3C028271	Plant transposase	Plant trans.	-	-	-	8.649
MELO3C031213	Gag/pol protein	Gag/pol	-	-	-	8.79
MELO3C034502	Gag/pol polyprotein	Gag/pol	-	8.544	-	-

Table S2. **Representative metabolites from *B. subtilis*-treated plants with identification parameters per Sumner et *al*., 2007[67].**

Proposed Compound Name	Leaf	$\log_2$ FC	IonMode	Adduct	Observed m/z	Calculated m/z	Ppm error
Rosmarinic acid	1	8.2188	Positive	$[M+H]^+$	343.1024	361.0918	56.4839
Caffeic acid	1 / 2	4.6769 / 3.0102	Positive	$[M+H]^+$	163.0391	181.0495	0.8423
Palatinose	1	3.4228	Positive	$[M+Na]^+$	325.0917	365.1060	59.3247
Abrine	1 / 2	4.4624 / 3.5593	Positive	$[M+H]^+$	188.0709	188.0708	4.7869
L-Tryptophan	1 / 2	6.5941 / 7.1977	Positive	M+H	205.0976	205.0972	2.9015
Protoporphyrin IX	1 / 2 / 3	-4.3462 / -5.548 / -3.5456	Positive	$[M+H]^+$	563.2659	563.2653	22.9727
PC-DAG (16:0/18:3)	2	4.5576	Positive	$[M+H]^+$	756.5539	756.5538	3.7917
Isoorientin	2	1.7423	Positive	$[M+H]^+$	449.1083	449.1078	1.0193

Table S3. **Representative metabolites from *B. velezensis*-treated plants with identification parameters per Sumner et *al*., 2007[67].**

Metabolite	Leaf	log_2 FC	IonMode	Adduct	Observed m/z	Calculated m/z	Ppm error
2-Linoleoyl-1-palmitoyl-sn-glycero-3-phosphoetanolamine	1 / 2	7.8944 / 5.987	Positive	$[M+H]^+$	716.5226	716.5232	0.59628
2-amino-5-(carbamoylamino)pentanoic acid (citrulline)	1	6.6909	Positive	$[M+H]^+$	176.1037	176.1034	3.9858
Isoorientin	1 / 2	5.4093 / 2.6828	Positive	$[M+H]^+$	449.1083	449.1088	1.0193
Isovitexin	1	4.524	Positive	$[M+H]^+$	433.1132	433.1133	0.5637
Arachidonoylthio-PC	1	4.4946	Positive	M+H	784.5885	784.5883	5.7567
PC-DAG (16:0/18:3)	1 / 2	4.4863 / 5.3095	Positive	$[M+H]^+$	756.5539	756.5538	3.7917
PE-DAG (16:1/18:2)	1 / 2	3.8627 / 3.8916	Positive	$[M+H]^+$	714.5075	714.5074	2.1356
Saponarin	1	3.281	Positive	$[M+H]^+$	595.166	595.1658	0.4102
Luteolin-7-glucoside	1	3.064	Positive	$[M+H]^+$	449.1084	449.1082	0.8834
Glutathione reduced	1	2.7304	Positive	$[M+H]^+$	308.0909	308.0908	0.2972
Protoporphyrin IX	2 / 3	-3.6758 / -2.8864	Positive	$[M+H]^+$	563.2659	563.2653	22.9727
1,2-Ditetradecanoyl-sn-glycero-3-phosphocholine	2 / 3	8.3261 / 8.7569	Positive	$[M+H]^+$	678.4735	678.4734	0.7197

L-Tryptophan	2 / 3	6.0517 / 6.18	Positive	M+H	205.0976	205.0972	2.9015
3-Indoleacrylic acid	2 / 3	4.3084 / 4.3484	Positive	$M+H-H_2O$	170.0604	170.0601	2.1534
Sinapic acid	2 / 3 / 4	2.207 / 3.2603 / 1.2376	Positive	$[M+H]^+$	207.0654	207.0653	1.1054
Ferulate	3	3.3913	Positive	$[M+H]^+$	195.0655	195.0652	2.5814
Abrine	3	2.8197	Positive	$[M+H]^+$	188.0709	188.0708	4.7869
Methyl trans-cinnamic acid	3	2.2938	Positive	$[M+H]^+$	163.0755	163.0754	3.0878
α-tocopherol	3	1.7172	Positive	$[M+H]^+$	430.3798	430.3796	0.4964
DL-Phenylalanine	3	1.5699	Positive	$[M+H]^+$	166.0865	166.0863	21.1311

Table S4. Primer sequences used in qRT-PCR analyses.

Gene	GeneID	Primers pairs	Sequence (5' → 3')	Amplicon (bp)
Pathogenesis-related protein PRB1-2 (*PR1-1a*)	103495329	CmPR1_F	GCATCAACGACTGTAGGCTAGT	117
		CmPR1_R	ACTGCTTCTCATTCACCCACAT	
Allene Oxide Synthase (*AOS*)	103487935	Cm_AOS_F	CGCTACGAGGCCATCTACAG	92
		Cm_AOS_R	CTTCAAAGATGCCACTGCCG	
Allene Oxide Cyclase (*AOC*)	103503379	Cm_AOC_F	GGTTCAGAACAAGCAGTGCG	121
		Cm_AOC_R	CGAAGGAGTCGTAACGGAGG	
Granule bound starch synthase 1 (*GBSS1*)	103482870	Cm_GBSS1_b_F	GCAAGTGCGAAGGTTGCTTT	88
		Cm_GBSS1_b_R	CAGGCAGATTCAGAAGGGCA	
Granule bound starch synthase 2 (*GBSS2*)	103486994	Cm_GBSS2_a_F	GACAGCAGATCGTGTGGTCA	80
		Cm_GBSS2_a_R	CATGTAAACCCCAGCCTCCC	
Sucrose synthase 2 (*SucS2*)	103494755	Cm_SucS2_b_F	CTCGATTTCCTTCGAGCCCA	113
		Cm_SucS2_b_R	GAAAGGTGCTCCTCAGCCTT	
Actin 7 *(act7)*	103485254	CmActin7_F	CACTGGTATTGTGCTGGATTC	98
		CmActin7_R	CAAGGTCCAAACGGAGAATG	
